# (8a*RS*)-8,8a-Dihydro­furo[3,2-*f*]indolizine-6,9(4*H*,7*H*)-dione

**DOI:** 10.1107/S1600536811027383

**Published:** 2011-07-13

**Authors:** Viktor Vrábel, Július Sivý, Łubomír Švorc, Peter Šafář, Štefan Marchalín

**Affiliations:** a Institute of Analytical Chemistry, Faculty of Chemical and Food Technology, Slovak University of Technology, Radlinského 9, SK-812 37 Bratislava, Slovak Republic 81237; b Institute of Natural Sciences, Faculty of Mechanical Engineering, Slovak University of Technologyy, Námestie slobody 17, SK-812 31 Bratislava, Slovak Republic 81231; c Institute of Organic Chemistry, Catalysis and Petrochemistry, Faculty of Chemical and Food Technology, Slovak University of Technology, Radlinského 9, SK-812 37 Bratislava, Slovak Republic 81237

## Abstract

The title compound, C_10_H_9_NO_3_, is a chiral mol­ecule with one stereogenic carbon atom, but which crystallizes as a racemate in the centrosymmetric space group *P*2_1_/*n*. The central six-membered ring of the indolizine moiety adopts a definite envelope conformation, while the conformation of the oxopyrrolidine ring is close to that of a flat-envelope with a maximum deviation of 0.352 (1) Å for the flap atom.

## Related literature

For properties of indolizine derivatives, see: Malonne *et al.* (1998 ▶); Medda *et al.* (2003 ▶); Sonnet *et al.* (2000 ▶); Campagna *et al.* (1990 ▶); Pearson & Guo (2001 ▶); Gupta *et al.* (2003 ▶); Teklu *et al.* (2005 ▶). For their role as synthetic targets for pharmaceuticals, see: Gubin *et al.* (1992 ▶); Ruprecht *et al.* (1989 ▶). For the synthesis of the title compound, see: Szemes *et al.* (1998 ▶). For metric comparison with related compounds, see: Pedersen (1967 ▶).
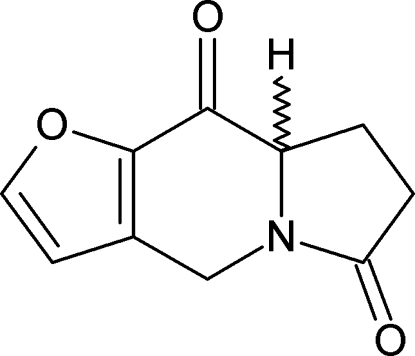


## Experimental

### 

#### Crystal data

C_10_H_9_NO_3_
*M* *_r_* = 191.18
Monoclinic, 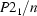

*a* = 7.63534 (19) Å
*b* = 11.7583 (2) Å
*c* = 9.9234 (3) Å
β = 105.775 (3)°
*V* = 857.35 (4) Å^3^
*Z* = 4
Mo *K*α radiation
μ = 0.11 mm^−1^
*T* = 298 K
0.49 × 0.23 × 0.13 mm

#### Data collection

Oxford Diffraction Gemini R CCD diffractometer
Absorption correction: analytical (Clark & Reid, 1995 ▶) *T*_min_ = 0.952, *T*_max_ = 0.992
14552 measured reflections
2213 independent reflections
1646 reflections with *I* > 2σ(*I*)
*R*_int_ = 0.018

#### Refinement

*R*[*F* ^2^ > 2σ(*F* ^2^)] = 0.040
*wR*(*F* ^2^) = 0.120
*S* = 1.03
2213 reflections
127 parameters
H-atom parameters constrained
Δρ_max_ = 0.20 e Å^−3^
Δρ_min_ = −0.16 e Å^−3^

### 

Data collection: *CrysAlis CCD* (Oxford Diffraction, 2006 ▶); cell refinement: *CrysAlis RED* (Oxford Diffraction, 2006 ▶); data reduction: *CrysAlis RED*; program(s) used to solve structure: *SHELXS97* (Sheldrick, 2008 ▶); program(s) used to refine structure: *SHELXL97* (Sheldrick, 2008 ▶); molecular graphics: *DIAMOND* (Brandenburg, 2001 ▶); software used to prepare material for publication: *enCIFer* (Allen *et al.*, 2004 ▶).

## Supplementary Material

Crystal structure: contains datablock(s) I, global. DOI: 10.1107/S1600536811027383/bg2407sup1.cif

Structure factors: contains datablock(s) I. DOI: 10.1107/S1600536811027383/bg2407Isup2.hkl

Supplementary material file. DOI: 10.1107/S1600536811027383/bg2407Isup3.cml

Additional supplementary materials:  crystallographic information; 3D view; checkCIF report

## Figures and Tables

**Fig. 1.**
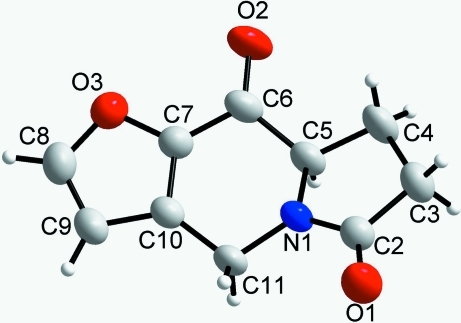
Molecular structure of (I) with the atomic numbering scheme; the chiral centre is C5. Displacement ellipsoids are drawn at the 50% probability level (Brandenburg, 2001).

**Fig. 2.**
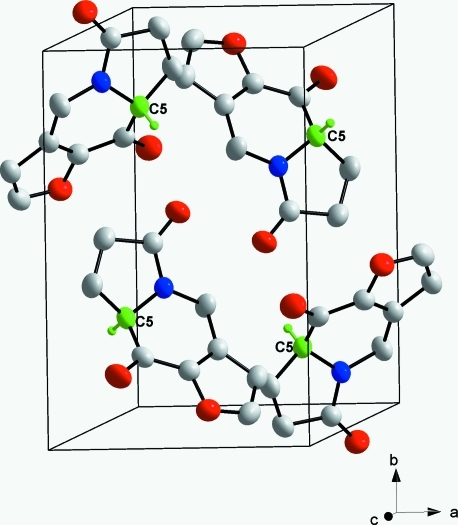
Packing diagram.

## supplementary crystallographic information

### Comment

Indolizine derivatives have been found to possess a variety of biological activities such as antiinflammatory (Malonne *et al.*, 1998), antiviral (Medda *et al.*, 2003), aromatase inhibitory (Sonnet *et al.*, 2000), analgestic (Campagna *et al.*, 1990) and antitumor (Pearson & Guo, 2001) activities. They have also shown to be calcium entry blockers (Gupta *et al.*, 2003) and potent antioxidants inhibiting lipid peroxidation *in vitro* (Teklu *et al.*, 2005). As such, indolizines are important synthetic targets in view of developing new pharmaceuticals for the treatment of cardiovascular diseases (Gubin *et al.*, 1992) and HIV infections (Ruprecht *et al.*, 1989).

Based on these facts and in continuation of our interest in developing simple and efficient route for the synthesis of novel indolizine derivatives, we report here the synthesis, molecular and crystal structure of the title compound, (I). The molecular structure and the atom labeling scheme are shown in Fig. 1.

The molecule crystallizes in the monoclinic space group P21/n. Accordingly, the compound is a racemate and consists of two enantiomeric pairs in the unit cell with relative configuration *R* and S on the C5 carbon atom. The central N-heterocyclic ring is not planar and adopts an envelope conformation for both enantiomers. A calculation of least-squares planes shows that this ring is puckered in such a manner that the five atoms C5, C6, C7, C10 and C11 are coplanar, while atom N1 is displaced from this plane with an out-of-plane displacement of 0.479 (2) Å.

The oxopyrrolidine ring attached to the indolizine ring system has a flat-envelope conformation, with atom C4 on the flap. The maximum deviation from planarity for C4 is 0.352 (1) Å. Obviously, the change of stereochemical centre on C5 from *R* to S causes changes in orientation of N1, C2, O1, C3 and C4.

The N1—C5 and N1—C11 bonds are approximately equivalent and both are much longer than the N1—C2 bond. Moreover, the N1 atom is *sp*^2^ hybridized, as evidenced by the sum of the valence angles around it [358.8 (3)° ]. These data are consistent with conjugation of the lone-pair electrons on N1 with the adjacent carbonyl and agree with literature values for simple amides (Pedersen, 1967).

### Experimental

The title compound rac-(8a)-8,8a-dihydrofuro[3,2-*f*]indolizine-6,9 (4*H*,7*H*)-dione was prepared according to a standard protocol described in literature (Szemes *et al.*, 1998).

### Refinement

All H atoms were placed in geometrically idealized positions and constrained to ride on their parent atoms, with C—H distances in the range 0.93 - 0.98Å and O—H distance 0.85Å and *U*_iso_ set at 1.2*U*_eq_ of the parent atom.

### Crystal data

**Table:**

C_10_H_9_NO_3_	*F*(000) = 400
*M**_r_* = 191.18	*D*_x_ = 1.481 Mg m^−^^3^
Monoclinic, *P*2_1_/*n*	Mo *K*α radiation, λ = 0.71073 Å
Hall symbol: -P 2yn	Cell parameters from 7934 reflections
*a* = 7.63534 (19) Å	θ = 3.5–29.3°
*b* = 11.7583 (2) Å	µ = 0.11 mm^−^^1^
*c* = 9.9234 (3) Å	*T* = 298 K
β = 105.775 (3)°	Block, yellow
*V* = 857.35 (4) Å^3^	0.49 × 0.23 × 0.13 mm
*Z* = 4	

### Data collection

**Table:**

Oxford Diffraction Gemini R CCD diffractometer	2213 independent reflections
Radiation source: fine-focus sealed tube	1646 reflections with *I* > 2σ(*I*)
graphite	*R*_int_ = 0.018
Detector resolution: 10.4340 pixels mm^-1^	θ_max_ = 29.4°, θ_min_ = 3.5°
Rotation method data acquisition using ω and φ scans	*h* = −10→10
Absorption correction: analytical (Clark & Reid, 1995)	*k* = −15→14
*T*_min_ = 0.952, *T*_max_ = 0.992	*l* = −13→13
14552 measured reflections	

### Refinement

**Table:**

Refinement on *F*^2^	Primary atom site location: structure-invariant direct methods
Least-squares matrix: full	Secondary atom site location: difference Fourier map
*R*[*F*^2^ > 2σ(*F*^2^)] = 0.040	Hydrogen site location: inferred from neighbouring sites
*wR*(*F*^2^) = 0.120	H-atom parameters constrained
*S* = 1.03	*w* = 1/[σ^2^(*F*_o_^2^) + (0.063*P*)^2^ + 0.130*P*] where *P* = (*F*_o_^2^ + 2*F*_c_^2^)/3
2213 reflections	(Δ/σ)_max_ < 0.001
127 parameters	Δρ_max_ = 0.20 e Å^−^^3^
0 restraints	Δρ_min_ = −0.16 e Å^−^^3^

### Special details

**Table:**

Experimental. face-indexed (Oxford Diffraction, 2006)
Geometry. All e.s.d.'s (except the e.s.d. in the dihedral angle between two l.s. planes) are estimated using the full covariance matrix. The cell e.s.d.'s are taken into account individually in the estimation of e.s.d.'s in distances, angles and torsion angles; correlations between e.s.d.'s in cell parameters are only used when they are defined by crystal symmetry. An approximate (isotropic) treatment of cell e.s.d.'s is used for estimating e.s.d.'s involving l.s. planes.
Refinement. Refinement of *F*^2^ against ALL reflections. The weighted *R*-factor *wR* and goodness of fit *S* are based on *F*^2^, conventional *R*-factors *R* are based on *F*, with *F* set to zero for negative *F*^2^. The threshold expression of *F*^2^ > σ(*F*^2^) is used only for calculating *R*-factors(gt) *etc*. and is not relevant to the choice of reflections for refinement. *R*-factors based on *F*^2^ are statistically about twice as large as those based on *F*, and *R*- factors based on ALL data will be even larger.

### Fractional atomic coordinates and isotropic or equivalent isotropic displacement parameters (Å^2^)

**Table:**

	*x*	*y*	*z*	*U*_iso_*/*U*_eq_	
C2	1.15109 (18)	0.99636 (12)	0.67889 (14)	0.0438 (3)	
C3	1.3182 (2)	1.01426 (13)	0.62848 (17)	0.0543 (4)	
H3B	1.4237	1.0285	0.7068	0.065*	
H3A	1.3015	1.0783	0.5647	0.065*	
C4	1.3418 (2)	0.90534 (14)	0.55443 (18)	0.0584 (4)	
H4B	1.4692	0.8843	0.5756	0.070*	
H4A	1.2938	0.9134	0.4538	0.070*	
C5	1.23351 (17)	0.81624 (11)	0.61138 (14)	0.0439 (3)	
H5A	1.3159	0.7767	0.6906	0.053*	
C6	1.13528 (19)	0.72915 (11)	0.50495 (14)	0.0456 (3)	
C7	0.96776 (18)	0.69128 (11)	0.53212 (13)	0.0429 (3)	
C8	0.7398 (2)	0.58131 (13)	0.53142 (16)	0.0539 (4)	
H8A	0.6557	0.5223	0.5095	0.065*	
C9	0.74188 (18)	0.66085 (12)	0.62999 (14)	0.0475 (3)	
H9A	0.6623	0.6671	0.6858	0.057*	
C10	0.89106 (17)	0.73291 (11)	0.63038 (13)	0.0408 (3)	
C11	0.96560 (19)	0.83359 (13)	0.71747 (14)	0.0483 (3)	
H11B	0.8692	0.8880	0.7146	0.058*	
H11A	1.0172	0.8106	0.8140	0.058*	
N1	1.10563 (14)	0.88532 (10)	0.66294 (11)	0.0425 (3)	
O1	1.07083 (16)	1.06761 (9)	0.72905 (13)	0.0636 (3)	
O2	1.19841 (17)	0.69185 (9)	0.41368 (12)	0.0654 (3)	
O3	0.87461 (14)	0.59746 (9)	0.46802 (10)	0.0535 (3)	

### Atomic displacement parameters (Å^2^)

**Table:**

	*U*^11^	*U*^22^	*U*^33^	*U*^12^	*U*^13^	*U*^23^
C2	0.0435 (6)	0.0436 (7)	0.0472 (7)	0.0004 (5)	0.0176 (5)	0.0013 (5)
C3	0.0515 (8)	0.0506 (8)	0.0679 (9)	−0.0066 (6)	0.0282 (7)	0.0042 (7)
C4	0.0544 (8)	0.0586 (9)	0.0760 (10)	−0.0066 (7)	0.0412 (8)	−0.0008 (7)
C5	0.0412 (6)	0.0457 (7)	0.0519 (7)	0.0039 (5)	0.0249 (6)	0.0033 (6)
C6	0.0541 (7)	0.0405 (7)	0.0511 (7)	0.0077 (6)	0.0294 (6)	0.0049 (5)
C7	0.0476 (7)	0.0408 (7)	0.0437 (7)	0.0003 (5)	0.0180 (6)	−0.0004 (5)
C8	0.0480 (8)	0.0501 (8)	0.0624 (9)	−0.0075 (6)	0.0131 (7)	0.0025 (7)
C9	0.0428 (7)	0.0526 (8)	0.0495 (7)	−0.0036 (6)	0.0169 (6)	0.0068 (6)
C10	0.0419 (6)	0.0441 (7)	0.0392 (6)	−0.0003 (5)	0.0161 (5)	0.0044 (5)
C11	0.0504 (7)	0.0550 (8)	0.0492 (7)	−0.0092 (6)	0.0301 (6)	−0.0073 (6)
N1	0.0428 (6)	0.0440 (6)	0.0487 (6)	−0.0026 (5)	0.0263 (5)	−0.0035 (5)
O1	0.0681 (7)	0.0486 (6)	0.0852 (8)	0.0021 (5)	0.0396 (6)	−0.0122 (5)
O2	0.0849 (8)	0.0569 (6)	0.0742 (7)	0.0039 (6)	0.0552 (7)	−0.0080 (5)
O3	0.0584 (6)	0.0480 (6)	0.0559 (6)	−0.0029 (4)	0.0188 (5)	−0.0085 (4)

### Geometric parameters (Å, °)

**Table:**

C2—O1	1.2211 (16)	C6—C7	1.4472 (18)
C2—N1	1.3492 (18)	C7—C10	1.3578 (17)
C2—C3	1.5062 (18)	C7—O3	1.3717 (16)
C3—C4	1.512 (2)	C8—C9	1.350 (2)
C3—H3B	0.9700	C8—O3	1.3579 (18)
C3—H3A	0.9700	C8—H8A	0.9300
C4—C5	1.5345 (19)	C9—C10	1.4188 (18)
C4—H4B	0.9700	C9—H9A	0.9300
C4—H4A	0.9700	C10—C11	1.4848 (19)
C5—N1	1.4651 (15)	C11—N1	1.4562 (15)
C5—C6	1.515 (2)	C11—H11B	0.9700
C5—H5A	0.9800	C11—H11A	0.9700
C6—O2	1.2176 (16)		
			
O1—C2—N1	124.75 (12)	C7—C6—C5	111.91 (10)
O1—C2—C3	127.14 (13)	C10—C7—O3	110.64 (11)
N1—C2—C3	108.08 (11)	C10—C7—C6	126.76 (13)
C2—C3—C4	105.48 (12)	O3—C7—C6	122.29 (11)
C2—C3—H3B	110.6	C9—C8—O3	112.18 (12)
C4—C3—H3B	110.6	C9—C8—H8A	123.9
C2—C3—H3A	110.6	O3—C8—H8A	123.9
C4—C3—H3A	110.6	C8—C9—C10	105.49 (12)
H3B—C3—H3A	108.8	C8—C9—H9A	127.3
C3—C4—C5	104.60 (11)	C10—C9—H9A	127.3
C3—C4—H4B	110.8	C7—C10—C9	106.58 (12)
C5—C4—H4B	110.8	C7—C10—C11	122.24 (11)
C3—C4—H4A	110.8	C9—C10—C11	131.16 (11)
C5—C4—H4A	110.8	N1—C11—C10	108.71 (10)
H4B—C4—H4A	108.9	N1—C11—H11B	109.9
N1—C5—C6	111.56 (10)	C10—C11—H11B	109.9
N1—C5—C4	103.12 (11)	N1—C11—H11A	109.9
C6—C5—C4	114.77 (12)	C10—C11—H11A	109.9
N1—C5—H5A	109.1	H11B—C11—H11A	108.3
C6—C5—H5A	109.1	C2—N1—C11	123.51 (10)
C4—C5—H5A	109.1	C2—N1—C5	113.76 (10)
O2—C6—C7	125.20 (14)	C11—N1—C5	121.64 (11)
O2—C6—C5	122.74 (13)	C8—O3—C7	105.10 (10)
			
O1—C2—C3—C4	−170.54 (15)	C8—C9—C10—C7	−0.07 (15)
N1—C2—C3—C4	11.15 (17)	C8—C9—C10—C11	−178.40 (15)
C2—C3—C4—C5	−20.30 (17)	C7—C10—C11—N1	9.17 (19)
C3—C4—C5—N1	21.70 (16)	C9—C10—C11—N1	−172.72 (13)
C3—C4—C5—C6	143.24 (13)	O1—C2—N1—C11	−6.8 (2)
N1—C5—C6—O2	152.99 (13)	C3—C2—N1—C11	171.60 (12)
C4—C5—C6—O2	36.17 (19)	O1—C2—N1—C5	−174.95 (14)
N1—C5—C6—C7	−31.17 (16)	C3—C2—N1—C5	3.41 (16)
C4—C5—C6—C7	−147.98 (12)	C10—C11—N1—C2	153.77 (12)
O2—C6—C7—C10	−178.15 (14)	C10—C11—N1—C5	−38.95 (17)
C5—C6—C7—C10	6.1 (2)	C6—C5—N1—C2	−139.84 (12)
O2—C6—C7—O3	9.0 (2)	C4—C5—N1—C2	−16.15 (16)
C5—C6—C7—O3	−166.75 (11)	C6—C5—N1—C11	51.73 (16)
O3—C8—C9—C10	−0.56 (16)	C4—C5—N1—C11	175.42 (12)
O3—C7—C10—C9	0.67 (15)	C9—C8—O3—C7	0.96 (16)
C6—C7—C10—C9	−172.91 (13)	C10—C7—O3—C8	−0.99 (15)
O3—C7—C10—C11	179.19 (12)	C6—C7—O3—C8	172.92 (13)
C6—C7—C10—C11	5.6 (2)		

## References

[bb1] Allen, F. H., Johnson, O., Shields, G. P., Smith, B. R. & Towler, M. (2004). *J. Appl. Cryst.* **37**, 335–338.

[bb2] Brandenburg, K. (2001). *DIAMOND* Crystal Impact GbR, Bonn, Germany.

[bb3] Campagna, F., Carotti, A., Casini, G. & Macripo, M. (1990). *Heterocycles*, **31**, 97–107.

[bb4] Clark, R. C. & Reid, J. S. (1995). *Acta Cryst.* A**51**, 887–897.

[bb5] Gubin, J., Lucchetti, J., Mahaux, J., Nisato, D., Rosseels, G., Clinet, M., Polster, P. & Chatelain, P. (1992). *J. Med. Chem.* **35**, 981–988. 10.1021/jm00084a002 1552511

[bb6] Gupta, S. P., Mathur, A. N., Nagappa, A. N., Kumar, D. & Kumaran, S. (2003). *Eur. J. Med. Chem.* **38**, 867–873. 10.1016/j.ejmech.2003.08.001 14575933

[bb7] Malonne, H., Hanuise, J. & Fontaine, J. (1998). *Pharm. Pharmacol. Commun.* **4**, 241–243.

[bb8] Medda, S., Jaisankar, P., Manna, R. K., Pal, B., Giri, V. S. & Basu, M. K. (2003). *J. Drug Target.* **11**, 123–128. 10.1080/1061186031000119101 12881199

[bb9] Oxford Diffraction (2006). *CrysAlis CCD* and *CrysAlis RED* Oxford Diffraction Ltd, Abingdon, England.

[bb10] Pearson, W. H. & Guo, L. (2001). *Tetrahedron Lett.* **42**, 8267–8271.

[bb11] Pedersen, B. F. (1967). *Acta Chem. Scand.* **21**, 1415–1424.

[bb12] Ruprecht, R. M., Mullaney, S., Andersen, J. & Bronson, R. (1989). *J. Acquir. Immune Defic. Syndr.* **2**, 149–157. 2495348

[bb13] Sheldrick, G. M. (2008). *Acta Cryst.* A**64**, 112–122. 10.1107/S0108767307043930 18156677

[bb14] Sonnet, P., Dallemagne, P., Guillom, J., Engueard, C., Stiebing, S., Tangue, J., Bureau, B., Rault, S., Auvray, P., Moslemi, S., Sourdaine, P. & Seralini, G. E. (2000). *Bioorg. Med. Chem.* **8**, 945–955. 10.1016/s0968-0896(00)00024-9 10882007

[bb15] Szemes, F., Marchalín, Š., Bar, N. & Decroix, B. (1998). *J. Heterocycl. Chem.* **35**, 1371–1375.

[bb16] Teklu, S., Gundersen, L. L., Larsen, T., Malterud, K. E. & Rise, F. (2005). *Bioorg. Med. Chem.* **13**, 3127–3139. 10.1016/j.bmc.2005.02.056 15809148

